# Interactome Networks of FOSL1 and FOSL2 in Human Th17 Cells

**DOI:** 10.1021/acsomega.1c03681

**Published:** 2021-09-16

**Authors:** Ankitha Shetty, Santosh D. Bhosale, Subhash Kumar Tripathi, Tanja Buchacher, Rahul Biradar, Omid Rasool, Robert Moulder, Sanjeev Galande, Riitta Lahesmaa

**Affiliations:** † Turku Bioscience Centre, University of Turku and Åbo Akademi University, Turku 20520, Finland; ‡ InFLAMES Research Flagship Center, University of Turku, Turku 20520, Finland; § Centre of Excellence in Epigenetics, Department of Biology, Indian Institute of Science Education and Research (IISER), Pune 411008, India; ∥ Protein Research Group, Department of Biochemistry and Molecular Biology, University of Southern Denmark, Odense M 5230, Denmark

## Abstract

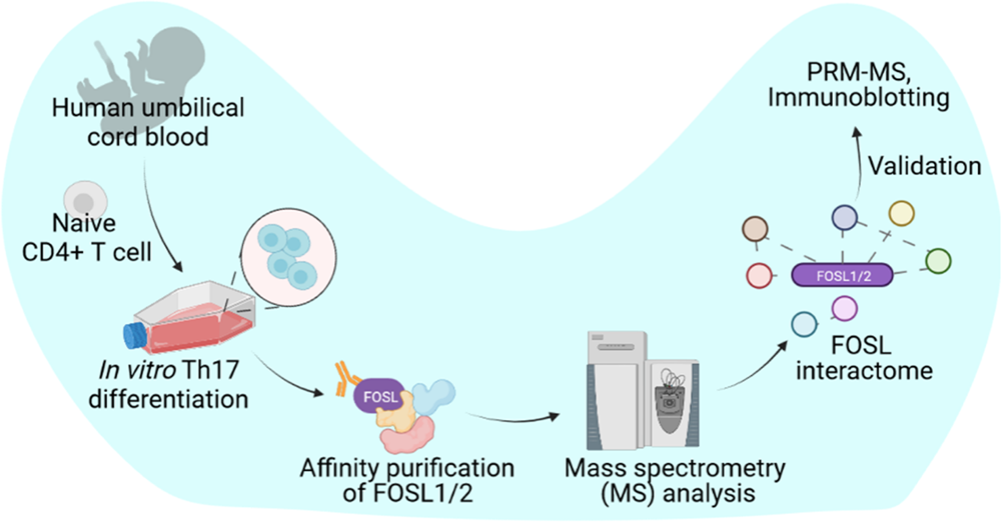

Dysregulated function of Th17 cells has implications in immunodeficiencies and autoimmune disorders. Th17 cell differentiation is orchestrated by a complex network of transcription factors, including several members of the activator protein (AP-1) family. Among the latter, FOSL1 and FOSL2 modulate the effector functions of Th17 cells. However, the molecular mechanisms underlying these effects are unclear, owing to the poorly characterized protein interaction networks of FOSL factors. Here, we establish the first interactomes of FOSL1 and FOSL2 in human Th17 cells, using affinity purification–mass spectrometry analysis. In addition to the known JUN proteins, we identified several novel binding partners of FOSL1 and FOSL2. Gene ontology analysis found a significant fraction of these interactors to be associated with RNA-binding activity, which suggests new mechanistic links. Intriguingly, 29 proteins were found to share interactions with FOSL1 and FOSL2, and these included key regulators of Th17 fate. We further validated the binding partners identified in this study by using parallel reaction monitoring targeted mass spectrometry and other methods. Our study provides key insights into the interaction-based signaling mechanisms of FOSL proteins that potentially govern Th17 cell differentiation and associated pathologies.

## 1 Introduction

Th17 cells are pro-inflammatory players that protect mucosal surfaces from extracellular pathogens. They can be derived *in vitro* by activating naive CD4^+^ T cells in the presence of IL-6, transforming growth factor-β (TGF-β), and interleukin (IL)-1β (or IL-23). These cells are mainly characterized by the expression of IL-17A and IL17F; however, they also secrete other cytokines, such as IL-21, IL-22, and GM-CSF.^1−6^ Deficiency of Th17 cells causes susceptibility to mucocutaneous candidiasis,^7^ whereas their uncontrolled activity can result in autoimmune conditions such as rheumatoid arthritis, multiple sclerosis, and systemic lupus erythematosus.^8^ To investigate the incidence of these associated diseases and design suitable therapeutic measures, it is crucial to first understand the molecular basis of Th17 cell function.

Th17 cell differentiation is initiated by the coordinated action of early expressed transcription factors (TFs), such as BATF, STAT3, and IRF4.^9^ This process is also modulated by members of the activator protein (AP-1) family, which includes the JUN (JUNB^10,11^), FOS (FOSL1,^12^ FOSL2^9^), and ATF (BATF,^13^ ATF3^14^) proteins. FOSL1 and FOSL2 (collectively termed FOS-like proteins) are two paralogous TFs that regulate embryonic development, cancer progression, and immune cell signaling.^15−18^ Their significance in initiating murine Th17 responses, however, was only recently realized.^9,12^ Though molecular networks are highly conserved in humans and mice, genetic studies across the two species have revealed striking discrepancies.^5,19−23^ In light of this, a parallel study from our laboratory used cord blood T cells to verify the human-specific roles of FOSL1 and FOSL2 in Th17-regulation.^24^ Functional genomics approaches revealed both of these factors to negatively influence Th17 responses in humans.^24^ Nonetheless, the molecular mechanisms that mediate these effects are not understood.

AP-1 TFs tend to bind to similar genomic sequences,^9,10,25^ but perform substantially different functions.^18^ Such versatility is attributed to their dynamic interactomes.^26−28^ FOSL1 and FOSL2 lack a transactivation domain and thus need to interact with JUN and other proteins to regulate their target genes. Furthermore, because these factors occupy DNA as a dimer, their regulatory abilities are significantly influenced by their interacting partners.^28^ Despite extensive research on AP-1 signaling, the interactomes of AP-1 TFs are largely unexplored in T cells. Mapping the interaction networks of FOSL proteins in human Th17 cells can thus advance our understanding of their signaling mechanisms in this milieu.

Affinity purification–mass spectrometry (AP–MS) has emerged as a reliable method for identifying protein–protein interactions (PPIs) at a global level.^29−31^ MS, in particular, detects and quantifies proteins in an unbiased manner, without prior knowledge. In the present study, we co-immunoprecipitated putative interactors of FOSL1 and FOSL2 in human Th17 cells and identified them by liquid chromatography with tandem MS (LC-MS/MS). Our analysis is the first to compare the FOSL1 and FOSL2 interactomes, thereby revealing their shared and unique binding partners. Parallel reaction monitoring targeted MS (PRM-MS) and immunoblotting (IB) were used to reliably validate the top interactors of these factors. Together with the predicted functionalities of the FOSL PPI networks, this study delivers a perspective on how FOSL proteins could regulate human Th17 cell identity. Such a comprehensive analysis could help gain insights into new therapeutic strategies for treating autoimmune diseases.

## 2 Results

### 2.1 FOS-like Proteins Are Upregulated during Initiation of Human Th17 Cell Differentiation

**Figure 1:**
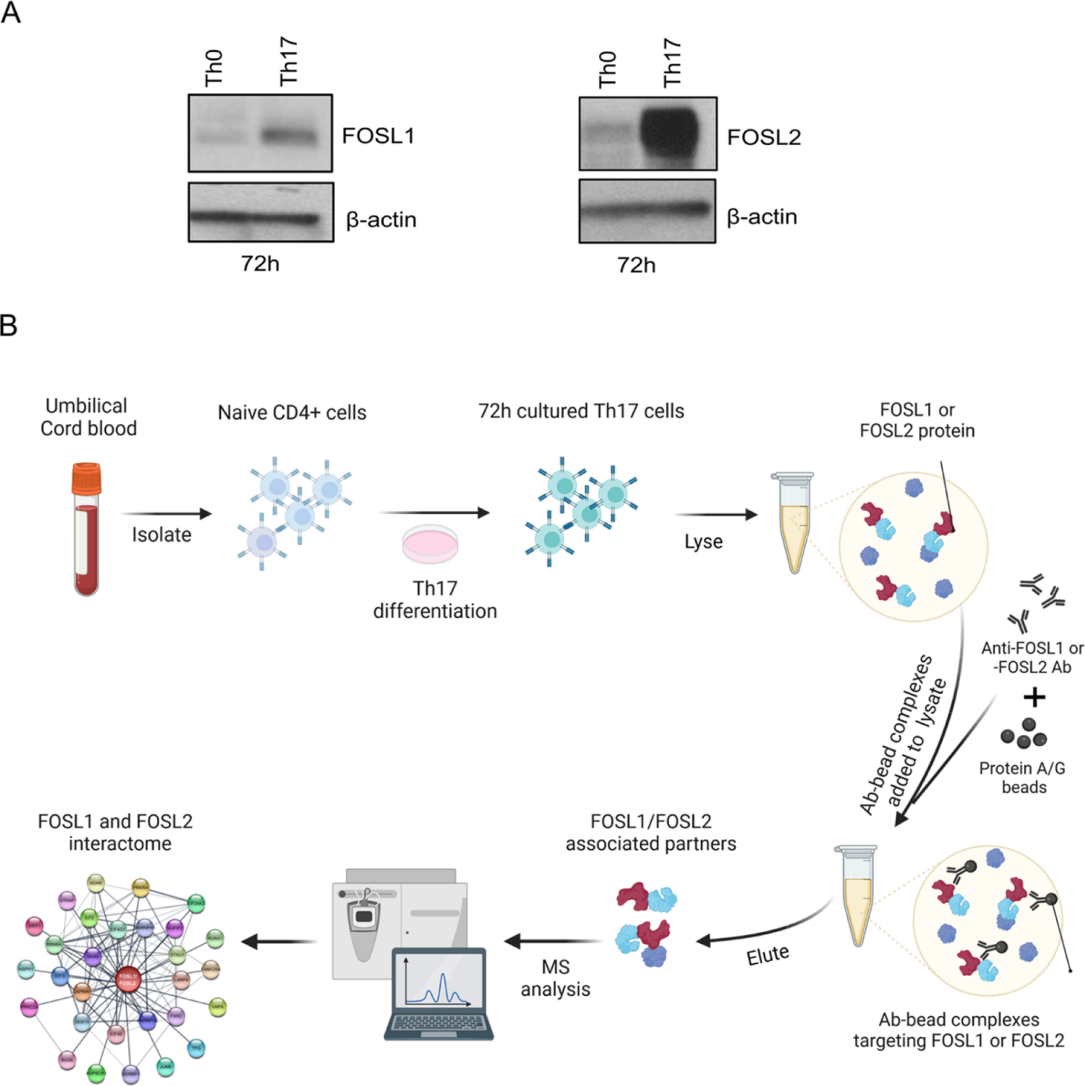
FOSL1 and FOSL2 expression and workflow for their proteomic analysis in human Th17 cells. (A) Immunoblots show expression of FOSL1 (left) and FOSL2 (right) in naive CD4^+^ T cells cultured under conditions of activation (Th0) or Th17-polarization for 72 h. Actin was used as the loading control. Blots for one of the three biological replicates are shown. (B) Workflow for the study. Naive CD4^+^ T cells were isolated from human umbilical cord blood and polarized to Th17 phenotype for 72 h. The cultured cells were lysed, and FOSL1 or FOSL2 protein was immunoprecipitated using their respective antibodies (Ab). The pull-down fractions were then analyzed for the binding partners of the respective factors using LC-MS/MS-based protein interactome analysis.

To study the interactomes of FOSL1 and FOSL2 in early Th17 cells, we first determined their expression in naive CD4^+^ T cells that were *in vitro* activated and differentiated toward Th17 fate for 72 h. This was the earliest time point where we reliably detected the expression of the Th17 differentiation markers, CCR6 and IL-17, using flow cytometry and enzyme-linked immunosorbent assay (ELISA), respectively (Figure S1A,B). Immunoblot analysis at this stage of differentiation also revealed a Th17-specific increase in the levels of FOSL1 and FOSL2, when compared to the activated (Th0) cells (Figures 1A and S1C). In light of the abovementioned results, we selected the 72 h time point for our proteomic analysis.

### 2.2 Systematic Analysis Unravels FOSL1 and FOSL2 Interacting Partners in Human Th17 Cells

**Figure 2:**
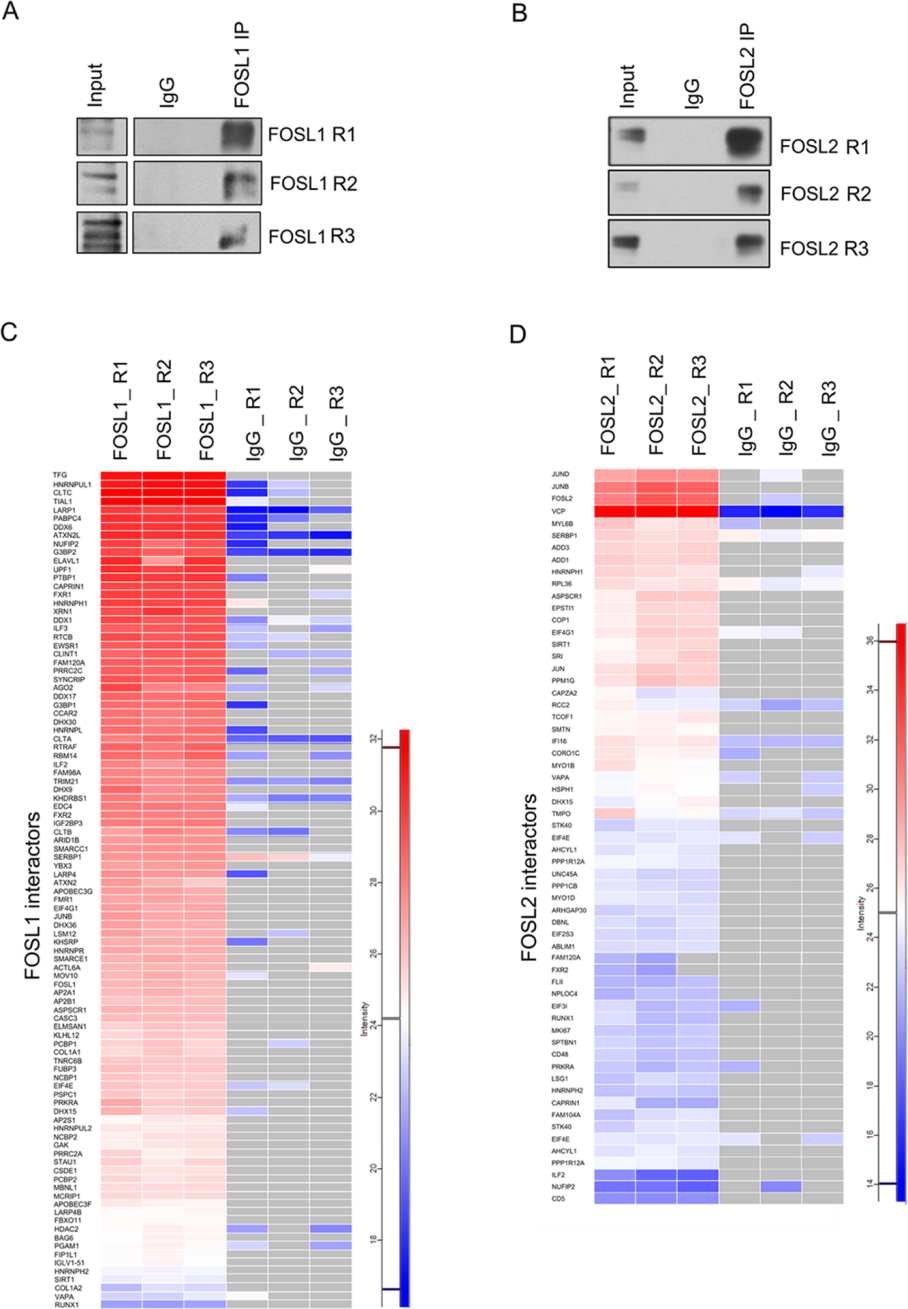
Analysis of FOSL1 and FOSL2 PPIs in human Th17 cells. (A,B) Immunoblots confirm immunoprecipitation of FOSL1 (panel A) and FOSL2 (panel B) protein from 72 h-cultured Th17 cell lysates. Blots show lanes for total lysate (input), IgG control IP, and FOSL1/FOSL2 IP. (C,D) FOSL1 or FOSL2 pull-down fractions from three biological replicates (R1, R2, and R3) were analyzed for their corresponding binding partners using LC-MS/MS. Heatmaps depict log_2_ intensity values for the topmost interacting partners of FOSL1 (panel C) and FOSL2 (panel D) in Th17 cells. Gray color indicates the missing or undetected proteins.

To identify the interacting partners of FOSL1 and FOSL2, an AP–MS approach was used. The workflow for the current study is illustrated in Figure 1B. Th17 cells from three independent biological replicates were lysed, and immunoprecipitation (IP) was performed using FOSL1 or FOSL2 Ab, as well as the species-specific control IgG Ab. Pull-down of the bait proteins (FOSL1 or FOSL2) was confirmed with IB (Figure 2A,B), and the IP fractions were then analyzed for interacting partners with LC-MS/MS. Using the MaxQuant label-free quantitation (LFQ) algorithm, the relative protein intensities were compared across the samples. The putative interactions were further prioritized by intensity, reproducibility, and specificity to the bait, with the mass spectrometry interaction statistics (MiST) algorithm.^32^ Our analysis reliably identified 163 and 67 binding partners of FOSL1 and FOSL2, respectively. These were obtained after strategically eliminating the nonspecific interactions, based on (1) comparing the enrichment scores with the IgG control and (2) using an in-house repository of possible contaminants derived from other AP–MS experiments in our laboratory.

The top binding partners of FOSL1 and FOSL2 and their corresponding enrichment scores are depicted in the heatmaps of Figure 2C,D (For all identified partners, see Figure S2A,B; Table S1). FOS–JUN dimers are one of the most widely occurring protein–protein associations across cell types. Amid members of the JUN family, JUNB was among the top interactors of both FOSL factors, which agrees with previous findings.^10,12,33^ Additionally, several new binding partners were identified. These included XRN1, AP2A1, PCBP1, ILF3, TRIM21, HNRNPH1, and HDAC2 for FOSL1 and ADD3, PPP1CB, MYO1B, HNRNPH1, CD48, and CD5 for FOSL2. Intriguingly, despite being paralogs with coordinated functions, FOSL1 and FOSL2 showed no interaction with each other. Although their association is reported in lower organisms, such as yeast,^34^ none of the existing studies in vertebrates support such findings.

### 2.3 Gene Ontology Functional Enrichment Analysis of FOSL1 and FOSL2 Interactors

**Figure 3:**
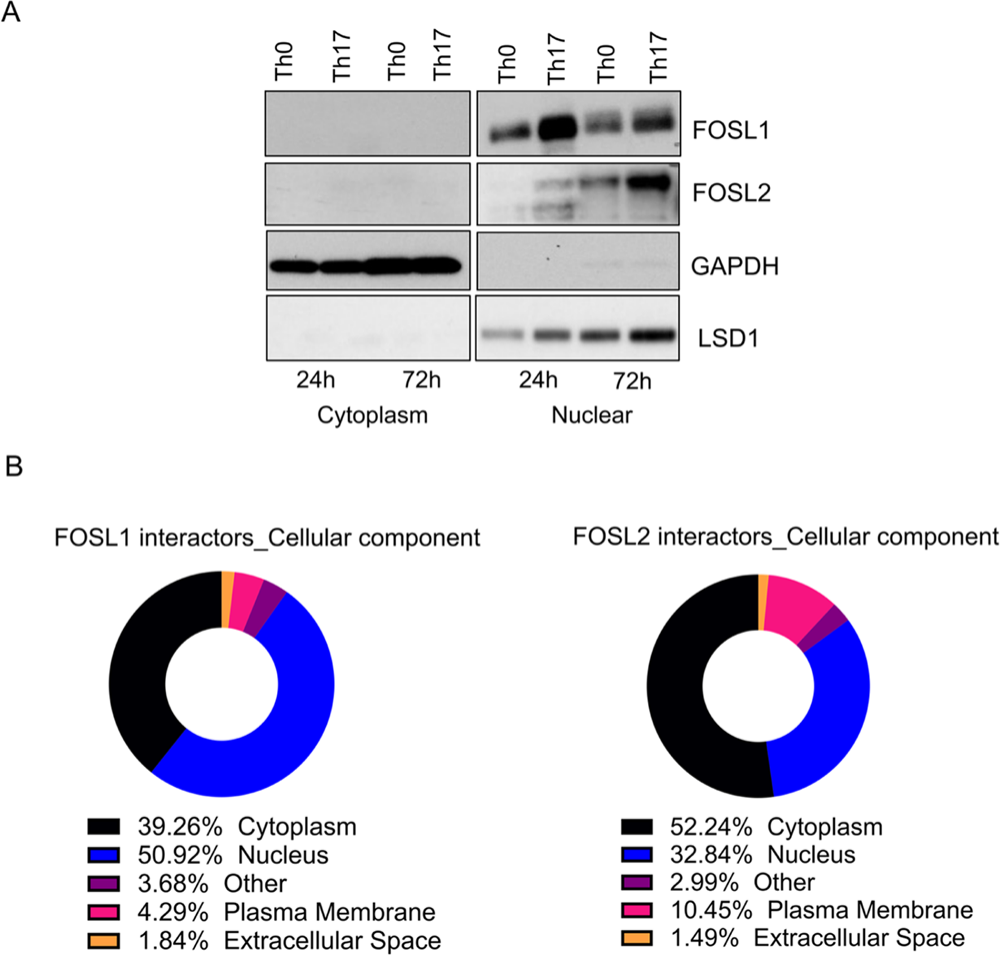
Localization of FOSL proteins and cellular distribution of their binding partners. (A) Following subcellular fractionation of Th0 and Th17 cell lysates (24 and 72 h), IB was performed to detect FOSL1 and FOSL2 in the fractionated samples. LSD1 and glyceraldehyde-3-phosphate dehydrogenase (GAPDH) were used to mark the nuclear and cytoplasmic fractions, respectively. Representative blot for three biological replicates is shown. (B) Pie charts showing classification of FOSL1 (left) and FOSL2 (right) interacting proteins based on their cellular localization, using IPA.

The spatial organization of signaling networks relies on the cellular location of the proteins that constitute the network.^35^ FOSL proteins can be cytoplasmic or nuclear and can shuttle between these compartments, in a context-dependent manner.^36,37^ Their localization profile in human T cells, however, is yet to be studied. We addressed this by performing subcellular fractionation on Th0 and Th17 lysates (24 and 72 h), which detected both proteins predominantly within the nuclear fractions (Figures 3A and S3A). To gain further insights into the FOSL-mediated signaling networks, the cellular distribution of their interacting partners was determined using Ingenuity pathway analysis (IPA) (Figure 3B; Table S3). More than 50% of the FOSL1 interactors and nearly 33% of the FOSL2 interactors were associated with the nucleus. Of the rest, most were cytoplasmic, and only a small fraction (10–15%) corresponded to other cellular compartments.

To determine the physiological relevance of these interactors, their molecular functionalities were mapped using the gene ontology (GO) database (Figure S4A,B; Table S4). Proteins interacting with FOSL1 were enriched for functions, such as RNA binding (60%; constituted by single-stranded, double-stranded, messenger RNA (mRNA), and small interfering RNA binding), nucleosomal DNA binding (6.67%), mRNA 5′ untranslated region binding (6.67%), RNA 7-methylguanosine cap binding (16.67%), clathrin binding (6.67%), and RNA helicase activity (3.33%) (Figure S4A). Similarly, FOSL2 interactors were enriched for translational initiation activity (66.67%), double-stranded RNA binding (16.67%), and actin filament binding (16.67%) (Figure S4B). Remarkably, RNA binding and translational initiation constituted more than 80% of the identified functionalities for either interactomes.

Previous studies have indicated the role of RNA-binding proteins (RBPs) in regulating FOS/JUN activity.^38−41^ RBPs are also known to modulate T cell development and function *via* post-transcriptional regulatory mechanisms.^42−45^ For instance, they can impair Th17 differentiation by destabilizing IL-17 RNA and other transcripts that code for Th17 regulatory factors.^46−51^ For degradation of the target mRNA, many RBPs coordinate with exonucleases such as XRN1.^50,52^ Interestingly, our analysis for FOSL1 interactome detected XRN proteins (XRN1 and XRN2) along with their known partners UPF1 and UPF2,^53^ which trigger mRNA decay.^54,55^ These findings imply that FOSL1 might restrain Th17 signaling by associating with proteins that destabilize the lineage-specific transcripts. This warrants further investigation.

**Figure 4:**
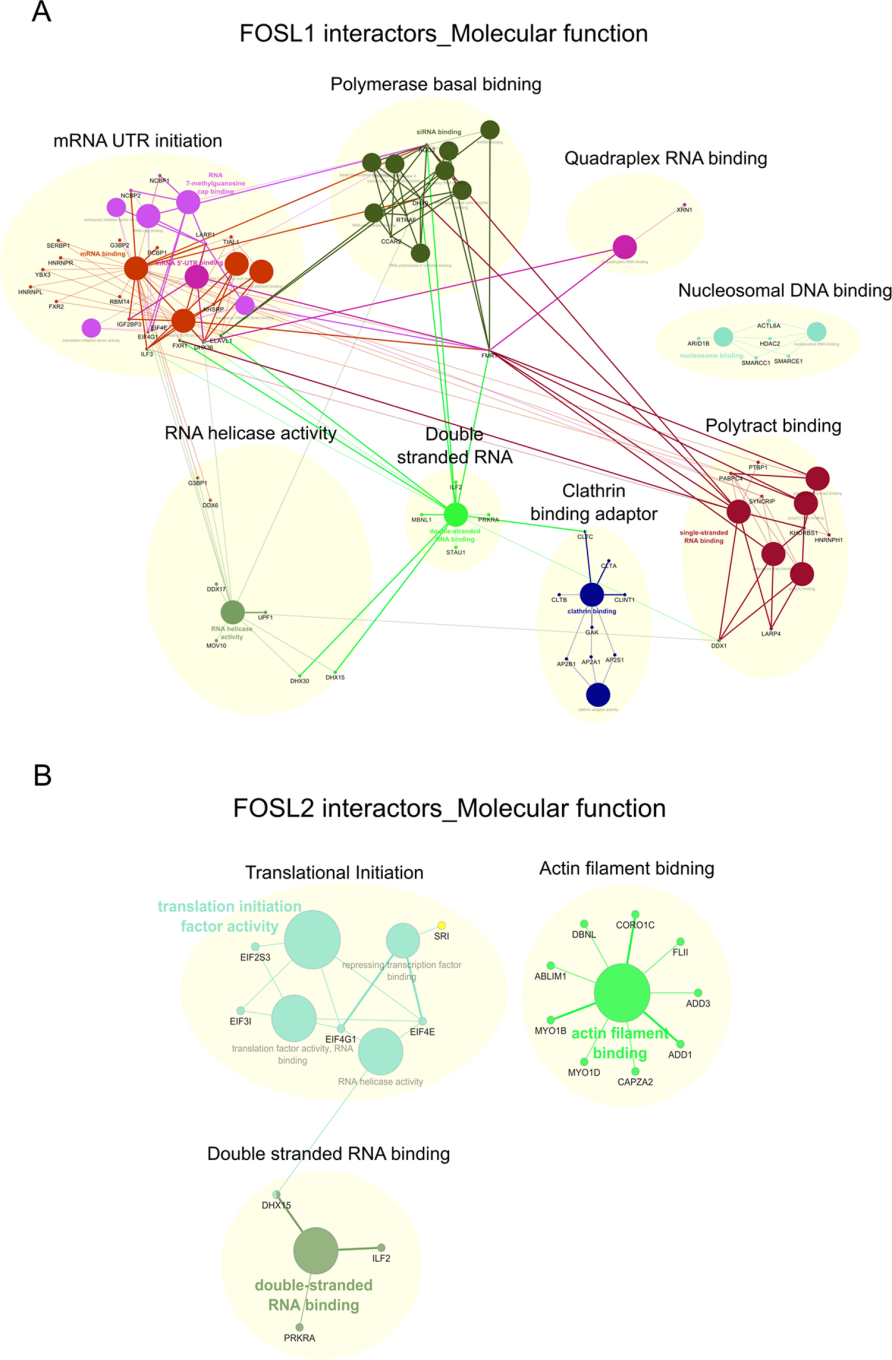
Molecular function networks enriched for FOSL1 and FOSL2 interactors. (A,B) FOSL1 (panel A) and FOSL2 (panel B) interactors were clustered based on their molecular functions and the resulting networks were visualized using ClueGO and CluePedia plugins built in Cytoscape (Bonferroni step-down corrected *p* values <0.05).

Network analysis was further performed for the enriched GO functionalities using Cytoscape (Figure 4A,B). Within the FOSL1 interaction network, the subclusters associated with different RNA-binding functions were highly interconnected. RBPs are also involved in the regulation of translational initiation through various mechanisms.^56^ This association was evident in the GO networks of both FOSL proteins (Figure 4A,B). Eukaryotic translational initiation factors (eIFs) stabilize ribosomal pre-initiation complexes and mediate post-transcriptional gene regulation.^56,57^ Within the eIF family, we found eIF4G1, eIF4E, eIF3I, and eIF2AK2 to interact with FOSL1, as well as FOSL2. Out of these, eIF4E constitutes the rate-limiting step for translational initiation by binding to the m7G cap of the transcripts.^57^ Interestingly, eIF4E is required for the pathogenesis of EAE in mice.^58^ It is also found to be targeted by the miRNA-467b in order to inhibit Th17 differentiation and autoimmune development.^59^ Thus, the binding of FOSL proteins to translational initiation factors points toward a unique strategy for monitoring Th17 responses.

### 2.4 FOSL1 and FOSL2 Share Interactions with Key Th17 Lineage-Associated Proteins

**Figure 5:**
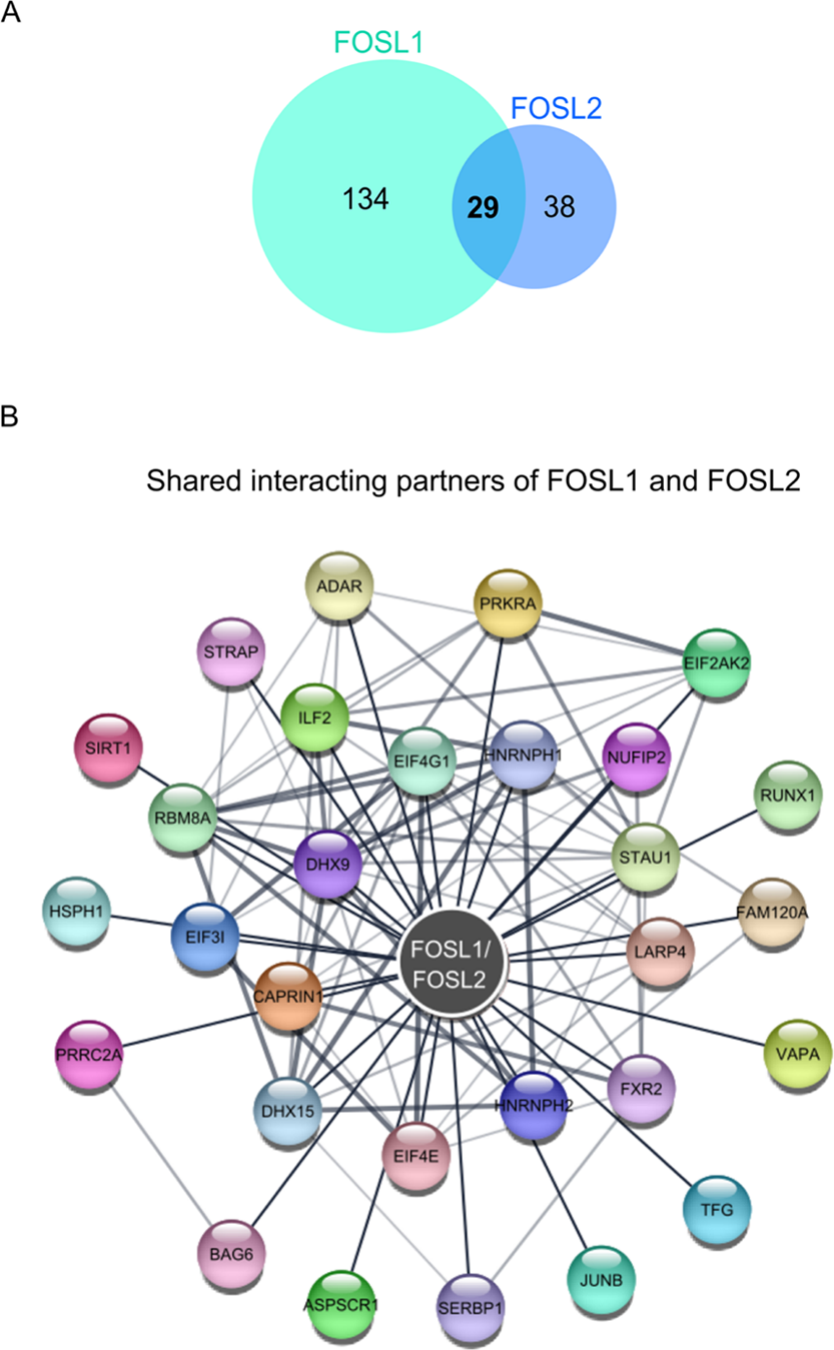
Shared interacting partners of FOSL1 and FOSL2 in human Th17 cells. (A) Venn diagram shows the number of shared and unique interactors of FOSL1 and FOSL2 in human Th17 cells, as identified by LC-MS/MS analysis. (B) PPIs shared between FOSL1 and FOSL2 were mapped against the STRING database and visualized using Cytoscape (a medium confidence score of 0.4 was used to create the STRING network).

Prediction models have indicated that interaction partners shared by candidate TFs could facilitate co-operative or competitive tendencies between the factors.^60^ Our recent study revealed a functional coordination between FOSL1 and FOSL2 during human Th17 regulation.^24^ To investigate whether an interactome-based mechanism regulates this paradigm, we analyzed these factors for their common binding partners. Our analysis revealed a total of 29 proteins to share interactions with FOSL1 and FOSL2, including JUNB, SIRT-1, HSPH1, DHX9, HNRNPH1/2, NUFIP2, LARP4, RUNX1, ADAR, and EIF4E, all of which are associated with T cell effector functions (Figure 5A,B; Table S2).

To study these common binding partners in the context of Th17 cell signaling, we focused on the ones that are known to regulate the lineage. These included JUNB,^10−12^ RUNX1,^61^ SIRT-1,^62^ eIF4E,^58,59^ and ADAR.^63^ Murine studies have found JUNB to promote Th17 fate, while restraining alternative lineages, by associating with BATF and FOSL2.^10^ Likewise, RUNX1 and SIRT-1 are reported to influence Th17 cell signaling. Interestingly, RUNX1’s effect on the lineage is largely governed by its binding partners. Its interaction with FOXP3 inhibits Th17 differentiation, whereas its association with RORγT activates the lineage.^61^ SIRT-1 analogously functions *via* RORγT, where it binds, deacetylates, and enables the latter to promote Th17 cell function.^62^ The abovementioned findings indicate that FOSL1 and FOSL2 may associate with the key regulators of the lineage in order to modulate effector responses of Th17 cells.

The list of common partners also included numerous proteins with undetermined roles in Th17 regulation, including NUFIP2, HNRNPH1, HNRNPH2, DHX9, DHX15, SERBP1, and others (Figure 5B). However, when evaluated in the context of other relevant studies, potential roles in controlling the Th17 lineage can be assigned to these factors. For instance, NUFIP2 acts as a co-factor for the RNA binding protein Roquin,^64^ which is reported to inhibit Th17 differentiation.^65^ This is possibly mediated *via* the post-transcriptional repression of Th17 activators, such as inducible T-cell COStimulator (ICOS),^64,66^ by the NUFIP2–Roquin complex. Our analysis also detected heterogeneous nuclear ribonucleoproteins (hnRNPs), namely, hnRNPH1 and hnRNPH2, which are involved in pre-mRNA alternative splicing. Interestingly, these proteins are closely associated with another member of the same family, hNRNPF, which reportedly interacts with FOXP3.^67^ Although FOXP3 is a master regulator of Treg differentiation, it also inhibits Th17 signaling by antagonizing RORγt.^68,69^ Thus, our MS analysis provides a number of new interacting partners that hint at novel mechanisms through which FOSL proteins alter Th17 cell fate.

### 2.5 Experimental Validation of the Shared and Unique Binding Partners of FOSL1 and FOSL2

**Figure 6:**
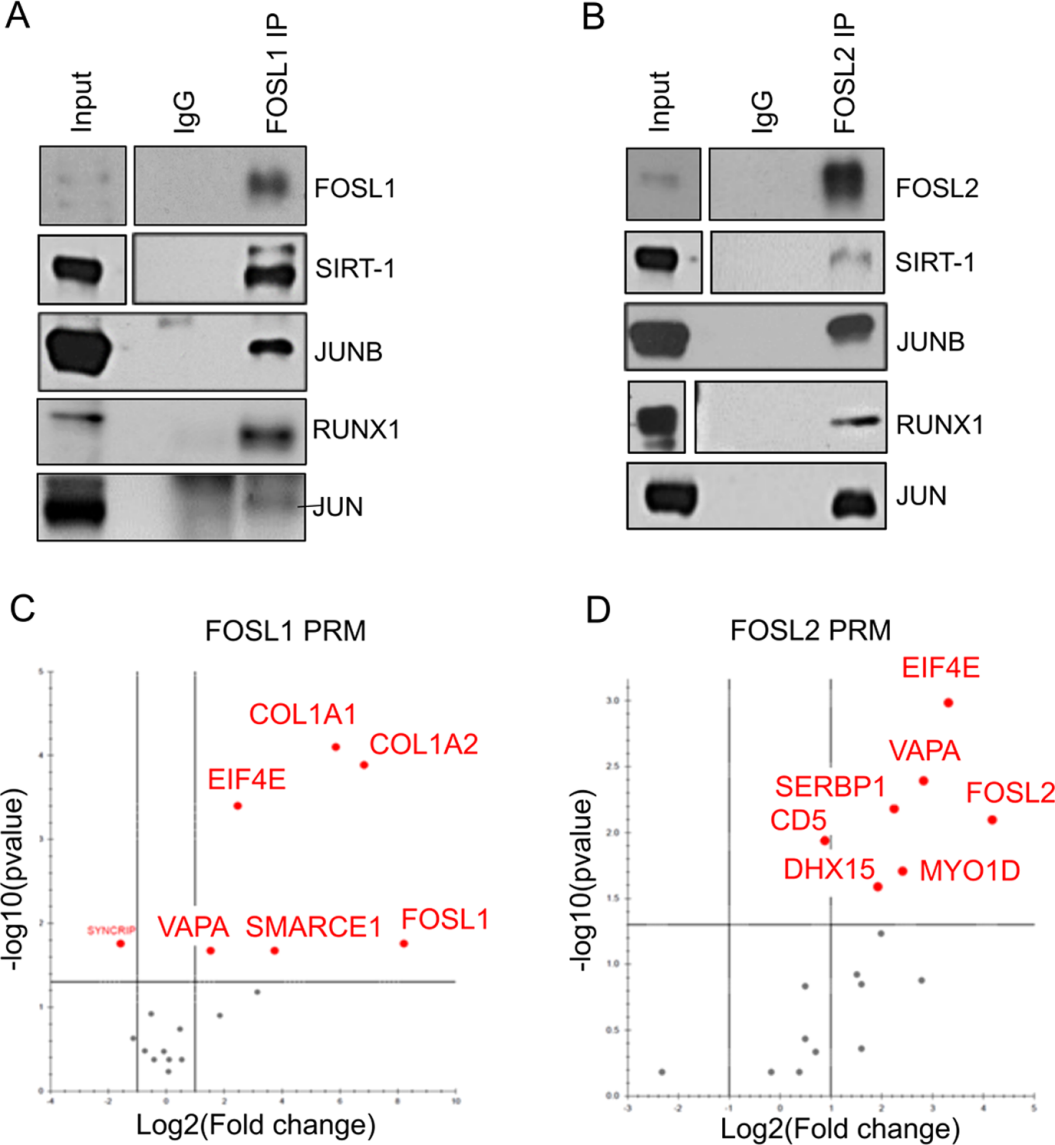
Validation of selected binding partners of FOSL1 and FOSL2 (A,B) FOSL1 (panel A) and FOSL2 (panel B) protein was immunoprecipitated from 72 h-cultured Th17 cells and western blotting was used to confirm their MS-identified interactions with SIRT-1, JUNB, RUNX1, and JUN. Blots depict lanes for total lysate (input), control IgG IP, and FOSL1/FOSL2 IP. Figures show representative blots for two or three biological replicates (see Figure S5 for all the replicates). (C,D) Volcano plots show selected binding partners of FOSL1 (panel C) and FOSL2 (panel D) that were validated by PRM-MS analysis. Data are representative of three biological replicates. The plots were extracted from Skyline.

To confirm their shared interactions with Th17-associated proteins, FOSL1 or FOSL2 was immunoprecipitated and immunoblotted to probe for JUNB, RUNX1, JUN, and SIRT-1 (Figures 6A,B and S5A,B). The results revealed a reproducible interaction of the FOSL factors with all of the assessed proteins. Although MS analysis of FOSL1 did not detect JUN, our IB findings indicate the presence of FOSL1–JUN complexes in Th17 cells.

Even among the unique interactors, we detected several candidates that have implications in Th17 development and inflammatory phenotypes. These included the FOSL1 partners α-1 type I collagen (COL1A1),^70^ SMARCE1,^71^ TRIM21,^72^ and HDAC2,^73^ as well as the FOSL2 interactors MYO1D,^74^ CD48,^75^ and JUND.^10^ A few of these, along with other FOSL-binding partners, were validated using targeted MS. With its increased sensitivity, reproducibility, and ease of implementation as a technique, PRM analysis was utilized to confirm selected interactions of FOSL1 (COL1A1, COL1A2, SMARCE1, VAPA, and EIF4E) and FOSL2 (SERBP1, DHX15, MYO1D, VAPA, and EIF4E) (Figure 6C,D).

Identification of FOSL1 binding to COL1A1 and COL1A2 was insightful because FOSL factors are known to regulate collagen production in other cell types.^76,77^ Additionally, changes in collagen protein levels are correlated with the development of rheumatoid arthritis and osteoarthritis.^70,78^ This suggests a potential involvement of FOSL1 in the incidence of human autoimmunity.

## 3 Discussion

AP-1 function is highly complex and is systematically regulated at multiple levels, such as, the choice of dimerizing partner, post-transcriptional/translational events, and additional interactions with bZIP or other unrelated proteins. Our recent functional genomics study found FOSL1 and FOSL2 to negatively regulate human Th17 differentiation.^24^ To decipher the mechanisms that govern such functions of FOSL proteins, we examined their binding partners using a whole-cell proteomics approach. Here, we report the first characterization of FOSL1 and FOSL2 interactomes in human Th17 cells. In addition to their known binding partners (JUN, JUNB, and JUND), our analysis identified many novel interactors of FOSL proteins. A significant fraction of these interactors appeared to be associated with RNA-binding functions. RBPs regulate gene expression by post-translationally modifying the stability and splicing of RNA molecules. Although previous studies have indicated RBP-mediated regulation of FOS activity,^38−41,54^ our findings for the first time holistically suggest a cross-talk between these protein groups. Further characterization on this line could broaden the horizons for AP-1 signaling mechanisms in Th17 cells.

Functionally synergistic TFs are known to bind over composite regulatory elements and this process involves physical interactions between different proteins to form regulatory complexes.^79^ Such mechanisms could be used to integrate distinct signaling pathways and create unified cellular responses. In our analysis, 29 proteins were found to share interactions with FOSL1 and FOSL2. Because both factors alter human Th17 differentiation in a similar fashion, their tendency to bind to common partners suggests functional cooperativity. Intriguingly, the shared hits included RUNX1, SIRT-1, and JUNB, all of which positively regulate Th17 differentiation in mice.^10,11,61,62,80^ If they have similar roles in the human counterpart, they could antagonize FOSL functions. Interaction-based mechanisms are commonly used by inhibitory proteins to dampen the activity of target TFs.^81,82^ In this respect, our findings suggest that FOSL proteins impair Th17 signaling by binding and sequestering the factors that support the lineage. Remarkably, JUNB and RUNX1 interact with both positive and negative regulators of Th17 fate, owing to which they can perform context-dependent functions. Additional mechanisms, such as post-translational modifications, differential expression profiles, and protein stability dynamics, may determine the outcome of their regulatory complexes.

A recent study by He et al. revealed interacting partners of FOSL1 in triple-negative breast cancer cells, many of which were reproducibly identified in our analysis (COL1A2, JUN, JUNB, CLTB, CLTC, FUBP3, KHDRBS1, RBM14, DDX17, HNRNPR, and XRN2).^83^ In addition, we found FOSL1 to associate with clathrin-binding adaptor proteins. This may be attributed to the non-endocytotic roles of clathrin, which involve its translocation to the nucleus to activate gene transcription.^84^ Furthermore, network analysis highlighted a link between the clathrin-binding cluster and double-stranded RNA binding. In relation to this, our study provides insights into the established role of clathrin-mediated endocytosis in the cellular uptake of pathogen-derived double-stranded RNAs.^85,86^ Follow-up experiments are required to determine the actual involvement of RBPs in this process.

FOSL1 was also observed to uniquely interact with factors such as histone deacetylase 2 (HDAC2) and poly-C binding protein 1 (PCBP1), which have reported roles in Th17 regulation. HDAC2 is a global modifier of gene expression that suppresses IL-17 transcription and thereby reduces colitis scores.^73^ In contrast, PCBP1 is a ferritin iron regulator that promotes Th17 pathogenicity and autoimmunity.^43,87^ These findings indicate that FOSL1 may control the lineage by associating with both the activator and repressor complexes. Other novel partners of FOSL1 included SWI/SNF family proteins (SMARCA2, SMARCB1, SMARCC1, SMARCC2, SMARCD2, and SMARCE1) and RNA helicase DEAD-box proteins (DDX6, DDX1). Interestingly, several members of these protein families are upregulated upon Th17 initiation,^20,71^ which hints at their involvement in development of the lineage.

The cytoskeleton plays an integral role in transducing extracellular signals to the nucleus.^88^ We found FOSL2 to interact with several proteins involved in actin filament binding (ADD1, ADD3, MYO1B, MYO1D, CAPZA2, ABLIM1, DBNL, CORO1C, and FLII). The depolymerization of actin or microtube networks is known to activate c-JUN function *via* the JNK/p38 signaling pathway.^89^ c-JUN expression is also induced by actin-binding proteins, such as profilin.^90^ Because JUN emerged as a shared interactor of FOSL1 and FOSL2 in our study, the findings mentioned above propose the involvement of cytoskeletal dynamics in regulating FOSL-mediated Th17 networks.

## 4 Conclusions and Outlook

In summary, this study uncovers, for the first time, the global binding partners of FOSL1 and FOSL2 in human T cells, with an emphasis on their shared interactors. Our analysis identified several novel PPIs and molecular functionalities as a part of FOSL-signaling networks. Moreover, the binding of key Th17 regulators to both FOSL1 and FOSL2 highlights the possible mechanisms that mediate the coordinated influence of these factors on the Th17 lineage. It is established that PPI networks of TFs are significantly altered in cases of mutations or disease.^91^ Because FOS-like proteins have important implications in the development of autoimmune disorders,^92−96^ their interactomes could serve as a crucial resource in the field of disease biology. Studying the changes in their PPIs under adverse physiological conditions could help predict diagnostic markers and therapeutic targets for Th17-associated pathologies.

## 5 Experimental Procedures

### 5.1 Human CD4^+^ T Cell Isolation

Mononuclear cells were isolated from human umbilical cord blood of healthy neonates (Turku University Central Hospital, Turku, Finland) by Ficoll-Paque density gradient centrifugation (Ficoll-Paque PLUS; GE Healthcare). Naive CD4^+^ T cells were further purified using CD4^+^ Dynal positive selection beads (Dynal CD4 positive isolation kit; Invitrogen), following the manufacturer’s protocol.

### 5.2 *In Vitro* Culturing of Th17 Cells

CD4^+^ T cells were activated with plate-bound α-CD3 (3.75 μg/mL; Immunotech) and soluble α-CD28 (1 μg/mL; Immunotech) in X-VIVO 20 serum-free medium (Lonza). X-VIVO 20 medium was supplemented with l-glutamine (2 mM, Sigma-Aldrich) and antibiotics (50 U/mL penicillin and 50 μg/mL streptomycin; Sigma-Aldrich). Th17 cell differentiation was induced using a cytokine cocktail of IL-6 (20 ng/mL; Roche), IL-1β (10 ng/mL), and TGF-β (10 ng/mL) in the presence of the neutralizing Ab anti-IFN-γ (1 μg/mL) and anti-IL-4 (1 μg/mL) to block Th1 and Th2 polarization, respectively. For the control cells (Th0), CD4^+^ T cells were TCR-stimulated with α-CD3 and α-CD28 in the presence of neutralizing Ab. All cytokines and neutralizing Ab were purchased from R&D Systems, unless otherwise stated. All cultures were maintained at 37 °C in a humidified atmosphere of 5% (v/v) CO_2_/air.

### 5.3 IL-17 Secretion

Secreted IL-17A levels were estimated in supernatants of 72 h-cultured Th17 cells using a human IL-17A DuoSet ELISA kit (R&D Biosystems DY317-05, DY008). The amount of IL-17A was normalized with the number of living cells based on forward and side scattering in flow cytometry analysis (LSRII flow cytometer; BD Biosciences).

### 5.4 Flow Cytometry

Th17 cells were harvested for 72 h, washed with flow cytometry staining buffer (0.5% fetal bovine serum; 0.1% Na-azide; and phosphate-buffered saline), and further incubated with PE-labeled anti-CCR6 antibody (BD cat no. 559562) for 20 min at 4 °C. Suitable isotype controls were used. Samples were analyzed using a LSRII flow cytometer (BD Biosciences). Live cells were gated based on forward and side scattering. The acquired data were analyzed with FlowJo (FlowJo, LLC).

### 5.5 Western Blotting

Cell culture pellets were lysed using radioimmunoprecipitation assay buffer (Pierce, cat no. 89901) that was supplemented with protease and phosphatase inhibitors (Roche) and sonicated using a Bioruptor UCD-200 (Diagenode). Sonicated lysates were centrifuged at 14,000 rpm for 30 min at 4 °C, and supernatants were collected. Samples were estimated for protein concentration (DC protein assay; Bio-Rad) and boiled in 6× Laemmli buffer (330 mM Tris-HCl, pH 6.8; 330 mM sodium dodecyl sulfate; 6% β-ME; 170 μM bromophenol blue; and 30% glycerol). Samples were then loaded on gradient Mini-PROTEAN TGX precast protein gels (Bio-Rad) and transferred to polyvinylidene fluoride membranes (Trans-Blot Turbo transfer packs, Bio-Rad).

For protein expression analysis of FOSL1 and FOSL2, the following Ab were used: anti-FOSL1 (Cell Signaling Tech., cat no. 5281), anti-FOSL2 (Cell Signaling Tech., cat no. 19967), and anti-β-actin (Sigma-Aldrich, cat no. A5441). HRP-conjugated anti-mouse IgG (Santa Cruz Biotechnology, cat no. sc-2005) and anti-rabbit IgG (BD PharMingen, cat no. 554021) were used as secondary Ab.

### 5.6 Cellular Fractionation

Cell pellets of Th0 and Th17 cultures (24 and 72 h) were lysed and fractionated into cytoplasmic and nuclear components using a NE-PER nuclear and cytoplasmic extraction reagent kit (Thermo Fisher Scientific, cat no. 78833) by following the manufacturer’s instructions. Extracts were then analyzed by western blotting. FOSL localization was determined using anti-FOSL1 (Cell Signaling Tech., cat no. 5281) and anti-FOSL2 (Cell Signaling Tech., cat no. 19967) Ab. Anti-GAPDH (HyTest, cat no. 5G4) and anti-LSD1 (Diagenode, cat no. C15410067) Ab were used to mark the cytoplasmic and nuclear fractions, respectively.

### 5.7 Immunoprecipitation

IP for FOSL1 and FOSL2 was performed using a Pierce MS-compatible magnetic IP kit (Thermo Fisher, cat no. 90409). Cell pellets from 72 h-cultured Th17 cells were lysed in appropriate volumes of lysis buffer (provided in the kit), which was supplemented with protease and phosphatase inhibitors (Roche). Lysates were estimated for protein concentration (DC protein assay; Bio-Rad). IP was performed using the following Ab: anti-FOSL1 (Santa Cruz Biotechnology, cat no. sc-28310), anti-FOSL2 (Cell Signaling Technology, cat no. 19967), mouse IgG (negative control for FOSL1: Cell Signaling, cat no. 5415), and rabbit IgG (negative control for FOSL2; Cell Signaling Technology, cat no. 2729). Equal amounts of Ab (μg) were used for each control IgG and FOSL IP reaction. All Ab were pre-incubated with 60 μL of protein A/G beads for 4–5 h to form antibody–bead complexes. Protein lysates (1 mg/IP reaction) were first pre-cleared with control IgG–bead complexes for 3 h. The pre-cleared lysates were then incubated overnight with FOSL1/FOSL2 antibody–bead complexes (test IP) or the corresponding control IgG–bead complexes (negative IP control). The pull-down fractions were washed (following the manufacturer’s protocol) and eluted with an elution buffer. Test and control IP samples were eluted in equal volumes of buffer. The eluted protein was vacuum-dried for MS analysis or run for western blotting.

The Ab used for IP–IB are as follows: anti-FOSL1 (Santa Cruz Biotechnology, cat no. sc-28310), anti-FOSL2 (Cell Signaling Technology, cat no. 19967), anti-RUNX1 A-2 (Santa Cruz Biotechnology, cat no. sc-365644); anti-JUNB C-11 (Santa Cruz Biotechnology, cat no. sc-8051); anti-SIRT1 (Cell Signaling Technology, cat no. 2496); and anti-JUN (BD Biosciences, cat no. 610326). Conformation-specific rabbit HRP (Cell Signaling Technology, cat no. 5127) and mouse HRP (Cell Signaling Technology, cat no. 58802) were used as secondary Ab.

### 5.8 Sample Preparation for Mass Spectrometry Analysis

The IP eluates for control IgG, FOSL1, and FOSL2 were denatured with urea buffer (8 M urea, 50 mM Tris-HCl, and pH 8.0), followed by reduction using dithiothreitol (10 mM) at 37 °C for 1 h. The reduced cysteine residues were subsequently alkylated using iodoacetamide (14 mM, in darkness) at room temperature for 30 min. The samples were diluted to reduce the urea concentration (<1 M), followed by digestion with sequencing grade modified trypsin at 37 °C overnight (16–18 h). The digested peptides were acidified and then desalted using C18 Stage Tips, prepared in-house using Empore C18 disks (3M, cat no. 2215). The desalted samples were dried in a SpeedVac (Savant SPD1010, Thermo Scientific) and then stored at −80 °C until further analysis.

For validation measurements, synthetic isotopic analogues (lysine ^13^C_6_^15^N_2_ and arginine ^13^C_6_^15^N_4_) were obtained for unique peptides from selected protein targets identified in the AP–MS discovery data (Thermo Fisher Scientific). The same sample preparation procedure was used for the validation experiments, with the exception that the samples were spiked with isotope-labeled peptides and MSRT retention time peptide standards (Sigma-Aldrich), prior to MS analysis.

### 5.9 LC-MS/MS Analysis

#### 5.9.1 Data-Dependent Analysis

The dried peptides were reconstituted in formic acid/acetonitrile (both 2% in water), and a NanoDrop-1000 UV spectrophotometer (Thermo Scientific) was used to measure the peptide amounts. Equivalent aliquots of the digested peptides were analyzed by LC-MS/MS using an EASY-nLC 1200 coupled to a Q Exactive HF mass spectrometer (Thermo Fisher Scientific). The peptides were loaded onto a 20 × 0.1 mm i.d. pre-column and separated with a 75 μm × 150 mm analytical column. The pre and analytical columns were packed with 5 and 3 μm Reprosil C18 respectively (Dr. Maisch GmbH). A separation gradient of 5–36% B in 50 min was used at a flow rate of 300 nL/min (solvent A: 0.1% formic acid in Milli-Q H_2_O and solvent B: 80% acetonitrile and 0.1% formic acid in Milli-Q H_2_O). The tandem MS spectra were acquired in a positive ion mode with a data-dependent Top 15 acquisition method at 300–1750 *m*/*z* using HCD fragmentation. The singly and unassigned charged species were excluded from the fragmentation. MS1 and MS/MS spectra were acquired in the Orbitrap, at a resolution set to 120,000 and 15,000 (at *m*/*z* 200), respectively. The AGC target values for MS1 and MS/MS were set to 3,000,000 and 50,000 ions, with maximal injection times of 100 and 150 ms, respectively, and the lowest mass was fixed at *m*/*z* 120. Dynamic exclusion was set to 20 s. Triplicate analyses were performed for all samples in randomized batches.

#### 5.9.2 Parallel Reaction Monitoring

Synthetic peptide analogues for validation targets (Table S5) were analyzed together with MSRT retention time peptide standards (Sigma-Aldrich) by LC-MS/MS using an Orbitrap Fusion Lumos mass spectrometer, coupled to an EASY-nLC (Thermo Fisher Scientific) with the same column configuration as above. On the basis of these data, a PRM method was developed for the analyses of these targets and their endogenous counterparts in AP validation samples. For the targeted analysis, the peptides were separated with a 30 min gradient of 8–39% solvent B. Data were acquired in a PRM mode with an isolation window setting of 1.6 *m*/*z* at a resolution of 15,000 for the Orbitrap, using a target AGC value of 50,000 and a maximum injection time of 22 ms.

### 5.10 Data Analysis

#### 5.10.1 AP–MS Data

The MS raw files were searched against a UniProt FASTA sequence database of the human proteome (downloaded, May 2019, 20415 entries) using Andromeda search engine, incorporated within MaxQuant software (Version 1.6.0.16).^97,98^ Trypsin digestion, with a maximum of two missed cleavages, carbamidomethylation of cysteine as a fixed modification, and variable modification of methionine oxidation and N-terminal acetylation were specified in the searches. A false discovery rate of 1% was applied at the peptide and protein levels. MaxQuant’s LFQ algorithm^99^ was used to calculate the relative protein intensity profiles across the samples. The “match between run” option was enabled to perform matching across the MS measurements.

The proteinGroup.txt file from the MaxQuant output was further processed using Perseus (Version 1.6.2.3).^100^ The output was filtered to remove contaminants, reverse hits, and proteins only identified by the site. Protein LFQ values were log_2_ transformed, and the medians of the technical replicates were calculated. The data were filtered to retain proteins with three valid values in at least one group (IgG, FOSL1, and FOSL2 pull-down). The resulting data matrix was then analyzed using the MiST algorithm. The algorithm calculates a MiST score for each of the potential interactors on the basis of their intensity, consistency, and specificity to the bait.^32^ MiST score criteria of ≥0.75 for FOSL1 and FOSL2-prey interaction and ≤0.75 for interaction with IgG were applied. Furthermore, to eliminate proteins frequently detected as contaminants in IP experiments, a comparison was made with a list of proteins that were detected with IgG mock baits in other T-helper cell studies of our laboratory (these were based on 126 other IP experiments). We retained those proteins that were detected with a frequency of less than 40% in the described in-house database for possible contaminants. Finally, we listed the top binding partners of FOSL1 and FOSL2 based on their abundance values in the respective FOSL IP, as compared to the corresponding IgG control.

Heatmaps for the subsequent list of FOSL1 and FOSL2 interactors, identified across three biological replicates (Pull-down *vs* IgG), were plotted using Perseus software. The gray color in the heatmaps represents the undetected proteins in the respective IP experiments. The interactors were additionally mapped against the STRING database, and the assigned PPI networks were further visualized using Cytoscape.^101^

#### 5.10.2 Validation Data

Data from analysis of the synthetic peptides were analyzed using Proteome Discoverer (Version 2.2, Thermo Fisher Scientific), and a FASTA file containing the sequences of the peptide targets. The MSF file from Proteome Discoverer was then used to construct a spectral library in Skyline (v4.2) software^102^ and define their retention time indices. Skyline was then used to create scheduled isolation lists for PRM analysis,^102^ process the PRM-MS raw files, and review the transitions and integration of the peptide peaks. The transition signals of endogenous peptides were normalized to their heavy counterparts, and the statistical analysis was performed using in-built MSstats plugin^103^ on the basis of sum of transition areas.

### 5.10.3 Data Availability

The mass spectrometry proteomics data have been deposited to the ProteomeXchange Consortium *via* the PRIDE^104^ partner repository with the data set identifier PXD025729. The details of PRM-MS measurements can be found in the Skyline Panorama^105^ link https://panoramaweb.org/FOSL1_2_Th17.url and are deposited to the ProteomeXchange Consortium *via* the PRIDE partner repository with the data set identifier PXD025840.

### 5.11 Cellular Component Analysis Using IPA

To map the cellular locations of the identified interactors, the list of binding partners was annotated using the IPA (www.qiagen.com/ingenuity; Qiagen; March 2019) tool.

### 5.12 GO Functional Enrichment Analysis and Networks

GO molecular function pie charts and networks were created using ClueGO and CluePedia plugins from Cytoscape based on the *p* value ≤0.05 and corrected using a Bonferroni step-down method.

### 5.13 Graphical Representation, Venn Diagrams, and Statistical Analysis

All graphs were plotted using GraphPad Prism software (V8.3.0). Two-tailed Student’s t test was used to calculate the statistical significance. Venn diagrams were generated using BioVenn.^106^

### 5.14 Graphical Illustration for Workflow of the Study

The pictorial representation for the workflow in Figure 1B was created using BioRender.com..

## Ethics Approval

The present study on primary human CD4^+^ T cells from the cord blood of neonates was conducted only after approval from the joint ethical committee of University of Turku and Turku University Hospital.

## Supporting Information Available

Th17 cell differentiation and associated expression of FOSL1 and FOSL2; FOSL1 and FOSL2 interacting proteins in human Th17 cells; subcellular fractionation of FOSL1 and FOSL2 in human Th17 cells; molecular functionalities of FOSL1 and FOSL2 interactors; and validation of shared interactors of FOSL1 and FOSL2 using immunoblot analysis (PDF)
List of FOSL1 and FOSL2 interactors; list of interactors shared between FOSL1 and FOSL2; IPA—cellular localization of FOSL1 and FOSL2 interactors; and gene ontology—molecular function analysis of FOSL1 and FOSL2 interactors; and list of synthetic peptides used in PRM analysis (XLSX)
