# Supplementary material for: Interactome Networks of FOSL1 and FOSL2 in Human Th17 Cells
Source: ACS Omega. 2021 Sep 16;6(38):24834–47. doi: 10.1021/acsomega.1c03681 (PMC8482465; doi:10.1021/acsomega.1c03681)
Supplement: Supplementary file 1 — ao1c03681_si_001.pdf [file ao1c03681_si_001.pdf]

SUPPLEMENTARY INFORMATION

The Interactome Networks of FOSL1 and FOSL2 in Human Th17 Cells

Ankitha Shetty[1, 2, 3, †], *Santosh D. Bhosale*[1, 4, †], *Subhash Kumar Tripathi*[1 †], *Tanja Buchacher*[1, 2], *Rahul Biradar*[1, 2], *Omid Rasool*[1, 2], *Robert Moulder*[1, 2], *Sanjeev Galande*[3], *and Riitta Lahesmaa*[1,2 *]

1 Turku Bioscience Centre, University of Turku and Åbo Akademi University; Turku 20520, Finland

2 InFLAMES Research Flagship Center, University of Turku

3 Centre of Excellence in Epigenetics, Department of Biology, Indian Institute of Science Education and Research (IISER); Pune 411008, India.

4 Protein Research Group, Department of Biochemistry and Molecular Biology, University of Southern Denmark, 5230 Odense M, Denmark.

* Corresponding author: rilahes@utu.fi

† These authors contributed equally

Table of contents

Figure S1. Th17 cell-differentiation and associated expression of FOSL1 and FOSL2

Figure S2. FOSL1 and FOSL2 interacting proteins in human Th17 cells

Figure S3. Subcellular fractionation of FOSL1 and FOSL2 in human Th17 cells

Figure S4. Molecular functionalities of FOSL1 and FOSL2 interactors

Figure S5. Validation of shared interactors of FOSL1 and FOSL2 using immunoblot analysis

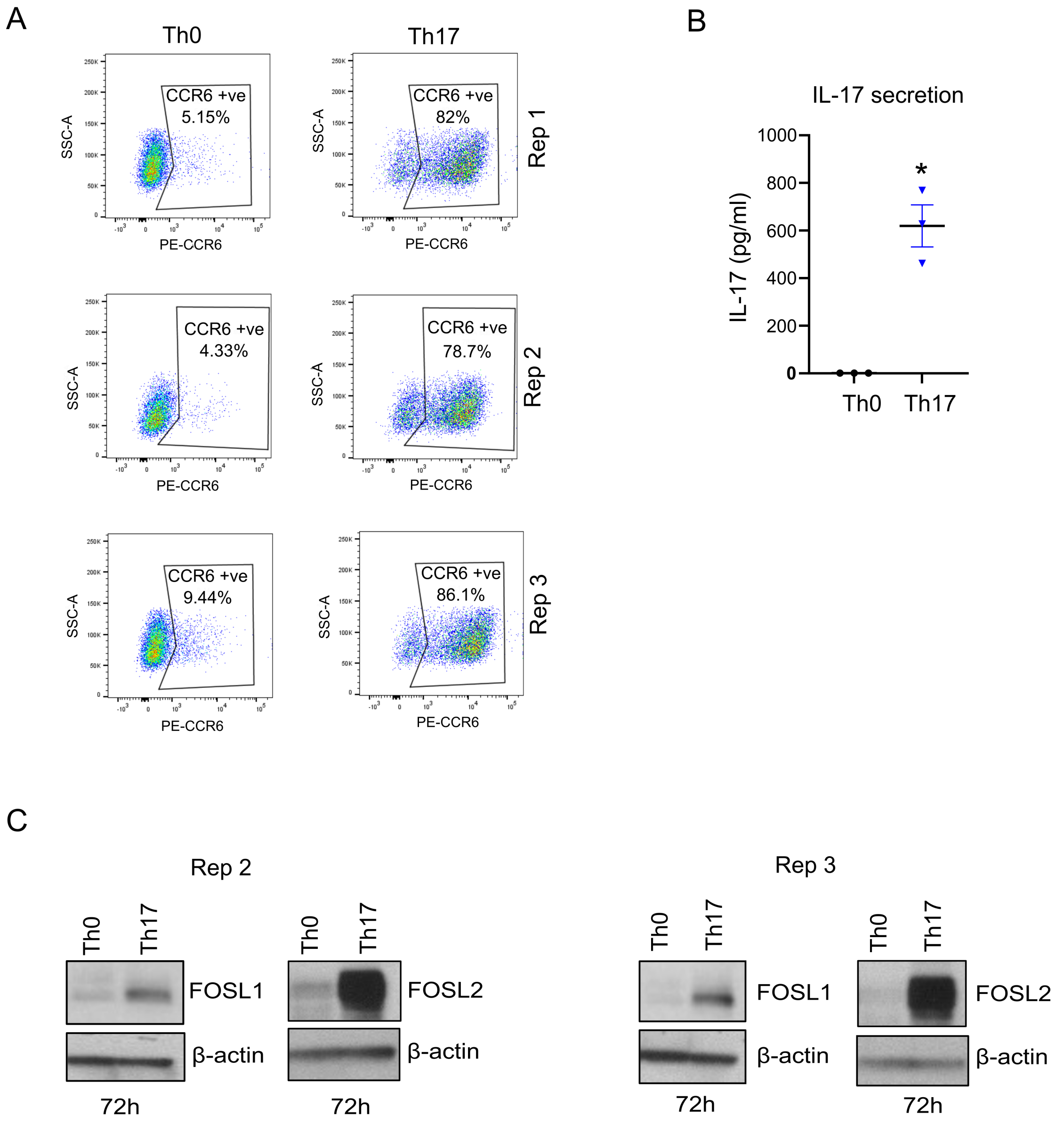

Figure S1. Th17 cell-differentiation and associated expression of FOSL1 and FOSL2

(A) Flow cytometry plots show percentage of CCR6 positive cells in Th0 and Th17 cultures at 72h of polarization. Cultures with polarization efficiencies equivalent to the ones shown here were used for proteomic analysis. Data for three biological replicates is shown. **(B)** Graph shows ELISA values for IL-17 secretion in 72h-cultured Th0 and Th17 cells. IL-17 values were first normalized to live cell count and then to Th0 control. Data is based on three biological replicates. Two-tailed students t-test was used to determine statistical significance

(*$p < 0.05$). **(C)** Immunoblots show protein levels of FOSL1 (left) and FOSL2 (right) in naive $CD4^+$ T cells cultured under activating (Th0) or Th17-polarizing conditions for 72h. Actin was used as loading control. Blots represent biological replicates for Figure 1A.

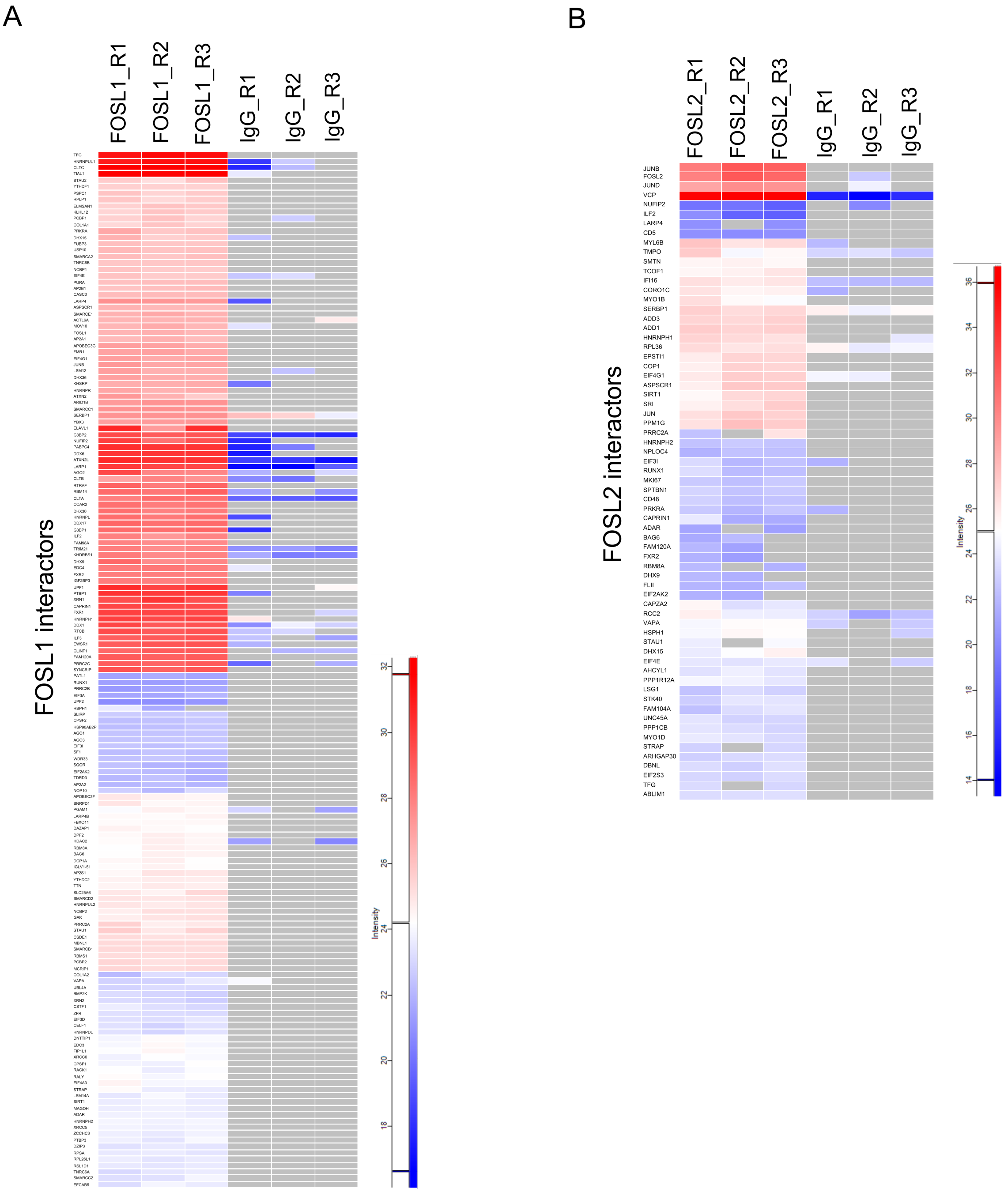

Figure S2. FOSL1 and FOSL2 interacting proteins in human Th17 cells

(A and B) Heatmaps shows Log_2intensity values for the FOSL1 (panel A) and FOSL2 (panel B) interactors that were identified by MS analysis in Th17 cells. Grey color represents undetected or missing proteins.

A

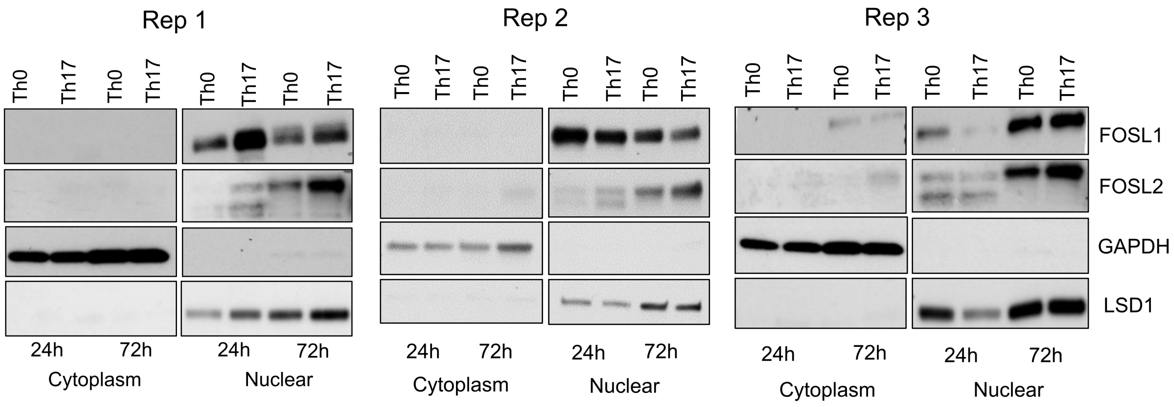

Figure S3. Subcellular fractionation of FOSL1 and FOSL2 in human Th17 cells

(A) Th0 and Th17 cell lysates (24h and 72h) were fractionated and further probed for expression of FOSL1 and FOSL2 using western blotting. GAPDH and LSD1 mark cytoplasmic and nuclear fractions, respectively. Rep 2 and 3 are biological replicates for the representative blot in Figure 3A.

A

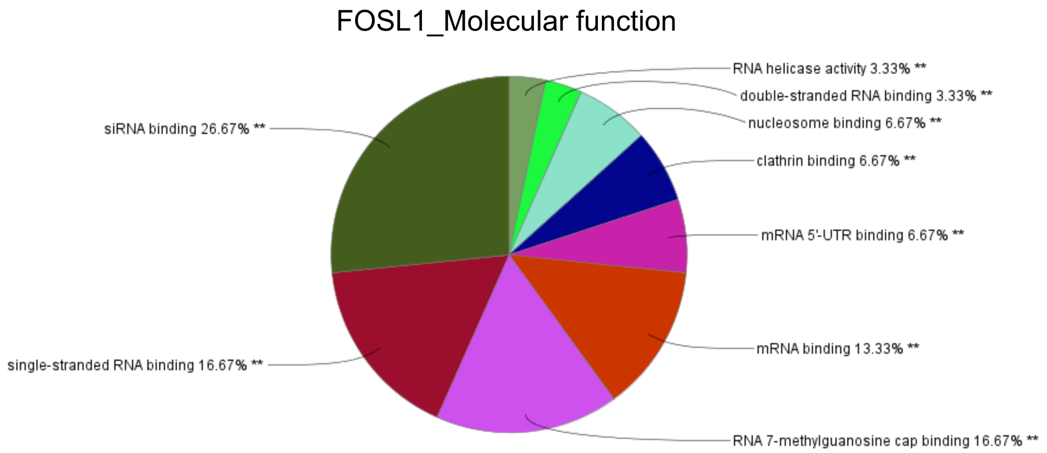

B

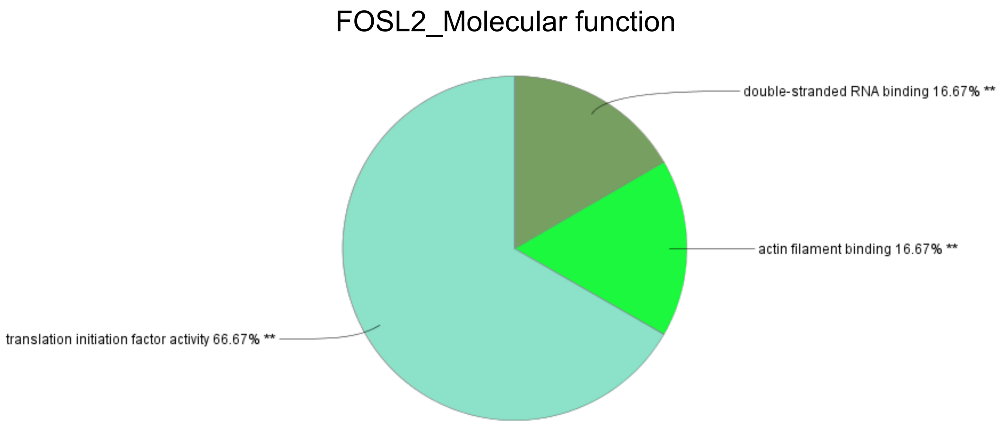

Figure S4. Molecular functionalities of FOSL1 and FOSL2 interactors

(A and B) Pie charts illustrate the classification of FOSL1 (panel A) and FOSL2 (panel B) interacting partners on the basis of their molecular function. ClueGO and CluePedia plugins from Cytoscape was used to create the charts.

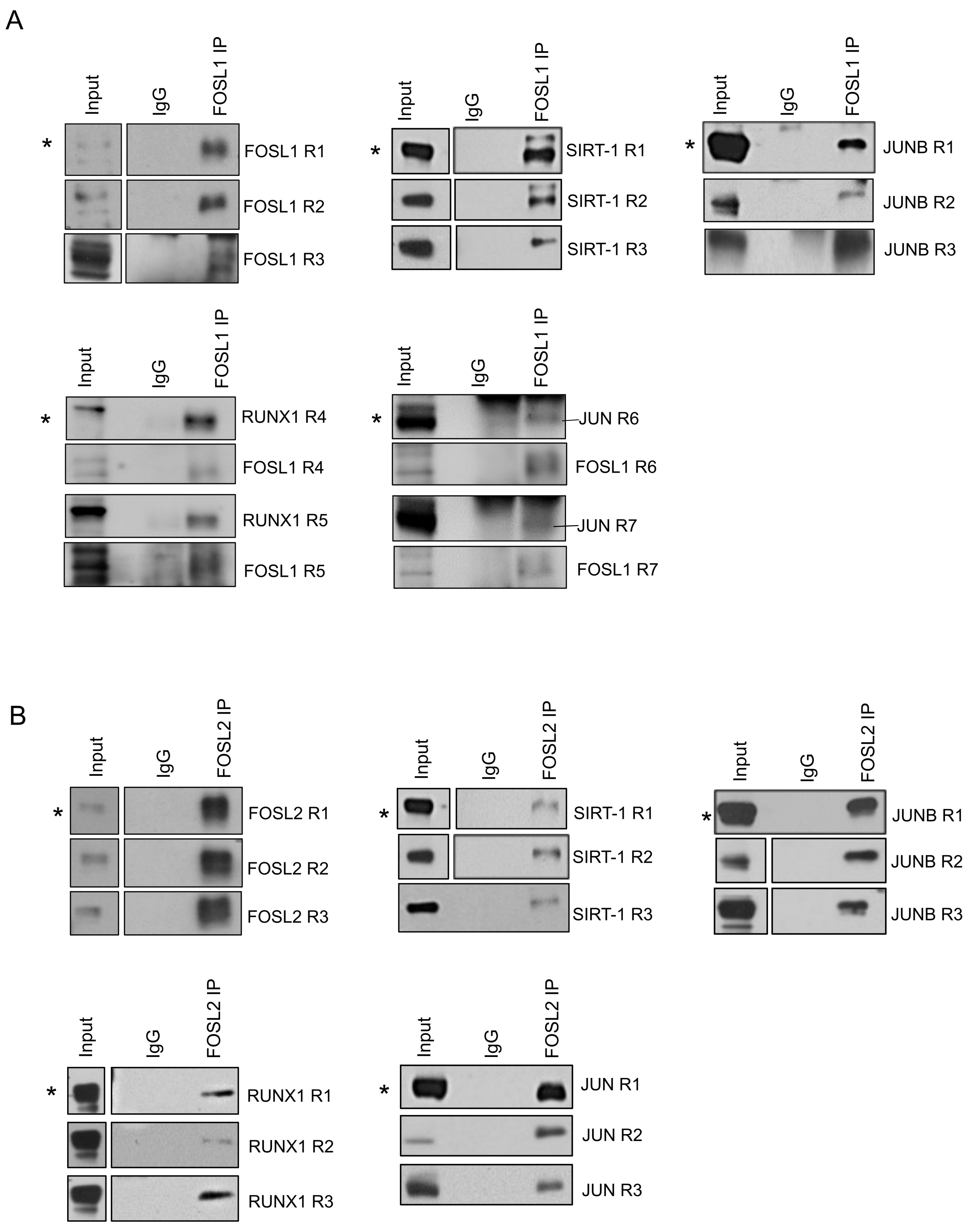

Figure S5. Validation of shared interactors of FOSL1 and FOSL2 using immunoblot analysis

(A and B) FOSL1 (panel A) or FOSL2 (panel B) protein was immunoprecipitated from 72h cultured Th17 cells and immunoblotting was performed to confirm their shared interactions with SIRT-1, JUNB, RUNX1 and JUN. Blots show lanes for total lysate (input), control IgG IP and FOSL1/FOSL2 IP. R1-R7 in panel A and R1-R3 in panel B represent different biological replicates for the FOSL1 and FOSL2 IP blots in Figs. 6A and B, respectively. The representative blots shown in Figure 6 are marked here with asterisk (*).
